# Acute Presentation of Newly Diagnosed Multiple Sclerosis Associated With Polymerase Chain Reaction-Proven Human Herpesvirus 6 Central Nervous System Infection

**DOI:** 10.7759/cureus.24319

**Published:** 2022-04-20

**Authors:** Conor M Pumphrey, Joshua F Scarcella, Donald L Price

**Affiliations:** 1 Medicine, Brody School of Medicine, Greenville, USA; 2 Plastic and Reconstructive Surgery, Brody School of Medicine, Greenville, USA; 3 Neurology, Vidant Medical Center/East Carolina University, Greenville, USA

**Keywords:** relapse, ms, multiple sclerosis, hhv-6, human herpesvirus 6

## Abstract

We present the case of a 26-year-old male who was found to have human herpesvirus 6 (HHV-6) in his cerebrospinal fluid (CSF) during acute presentation of multiple sclerosis (MS). Paresthesia of the lower extremities was his only symptom during the initial presentation, and workup for MS was not included during this evaluation. A single dose of IV steroids failed to improve his condition, and symptoms became more severe. Upon secondary evaluation, MRI revealed white-matter disease with plaques at multiple levels of the cervical spine and central nervous system (CNS). Lumbar puncture was obtained, and CSF analysis was positive for HHV-6 DNA. After five days of oral steroid treatment and physical therapy for three weeks, his symptoms continued to worsen. MRI at this time demonstrated an increase in the size of previous plaques and new foci of white matter disease. Repeat CSF analysis was negative for HHV-6. The virus’ association with relapse of MS has been investigated by many studies. However, there is a lack of literature investigating its role in causing MS disease. In this case report, we highlight the need for further research aimed at determining if HHV-6 is an environmental trigger for MS disease onset.

## Introduction

A link between the pathogenesis of multiple sclerosis (MS) and human herpesviruses has been thought to exist for decades. One of the earliest studies suggested that human herpesvirus 6 (HHV-6) has a higher association with MS than any other virus. Results showed that over 70% of MS and control brains contained HHV-6 DNA by polymerase chain reaction (PCR). However, immunocytochemistry found viral proteins within the oligodendrocytes of 80% of MS brains compared to 0% of control brains [1]. One of the most prominent explanations supporting a potential relationship between HHV-6 and MS is based on the concept of molecular mimicry. There is an association between T-cell reactivity to myelin basic protein (MBP) and infection with HHV-6 because the U24 antigen shares the same amino base sequence as MBP in part of the peptide [2]. Additionally, one study revealed that interleukin-2A and interleukin-7A receptor alleles predispose patients to the development of MS [3].

The role that HHV-6 plays in the onset of MS disease has yet to be defined. We present a case of MS notable for cerebrospinal fluid (CSF) analysis that was positive for HHV-6 DNA at the time of diagnosis. 

## Case presentation

A 26-year-old male was evaluated at a rural acute care hospital for bilateral lower extremity paresthesia. The patient received a dose of IV steroids for peripheral neuropathy of undetermined origin and was discharged home. Workup was limited to a complete blood count and a comprehensive metabolic panel. He returned a week later endorsing new paresthesia in his arms bilaterally. He received an MRI and a lumbar puncture with subsequent transfer to a larger acute care facility. A physical exam showed mild bilateral lower extremity strength deficits on resisted flexion and extension at the hip and knee joints. Sensory deficits to light touch were present in the right upper and bilateral lower extremities in a distribution not consistent with individual dermatomes. A positive Babinski sign was present on the right and a physical exam revealed cerebellar ataxia impairing the finger-to-nose test on the left and causing a wide-based gait. He did not demonstrate nystagmus or impaired ocular movement, and cranial nerves were intact to sensation and motor function tests. MRI results demonstrated an aggressive demyelinating disease burden. The patient was diagnosed with MS and received a five-day course of IV steroids. Lumbar puncture CSF collections at the time of diagnosis consisted of total protein levels within normal limits, normal white blood cell levels, elevated glucose, and meningitis/encephalitis PCR that was positive only for HHV-6. He was discharged home with a referral to physical therapy and a prednisone taper. The patient was ambulating without need for assistance, but several days before admission to our service, his condition worsened.

**Figure 1:**
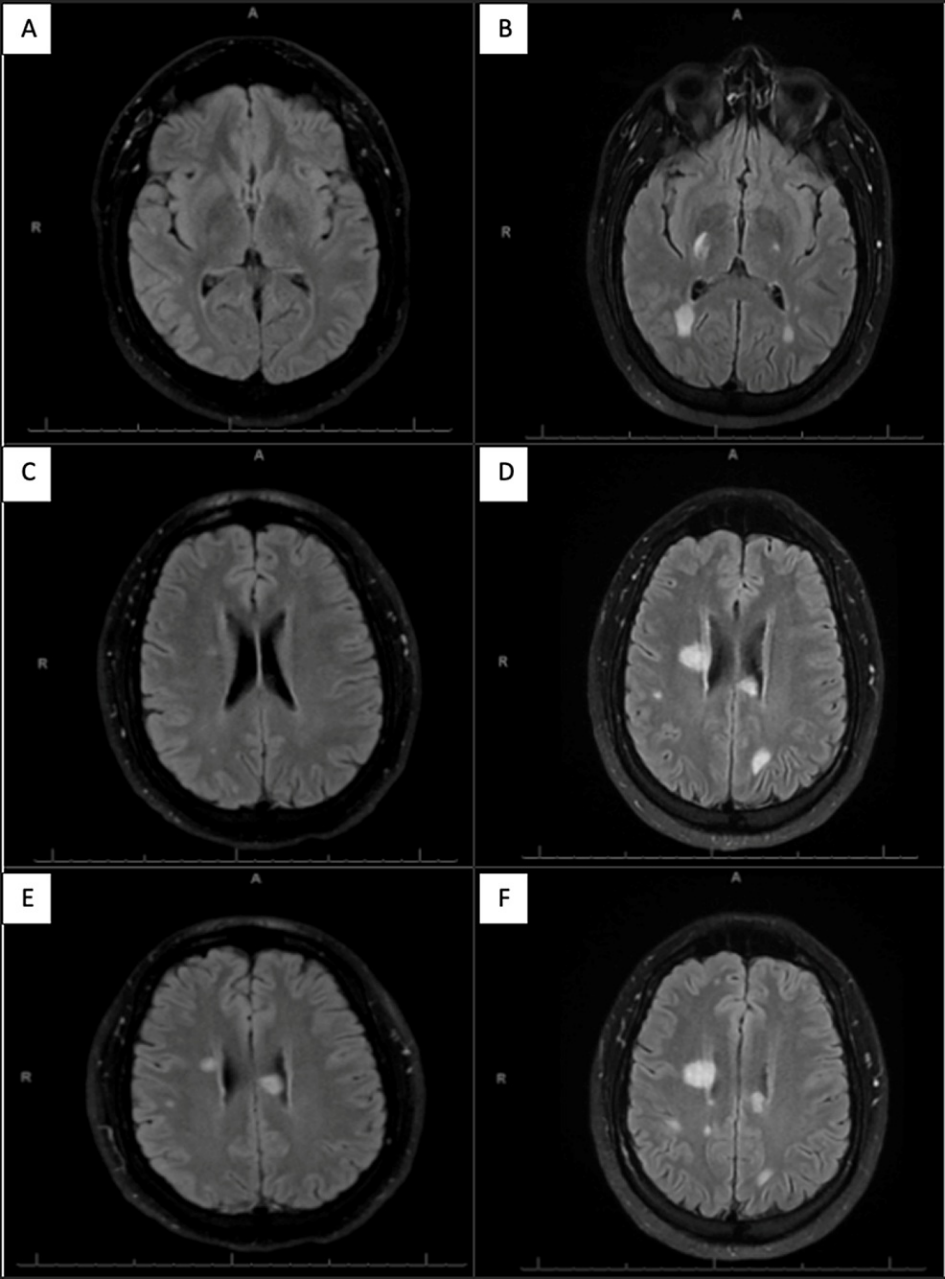
T2 Axial MRI Images T2 axial MRI images were taken on 5/7/21 at an outside hospital (A,C,E) and 5/31/2021 at our hospital (B,D,F) which show increased contrast uptake in focal white matter lesions and progressive disease. Figure A demonstrates an absence of plaque burden at this level during acute presentation and figure B shows four new lesions at the same level. Figure C shows a small right periventricular plaque while Figure D demonstrates a significant enlargement of previous plaque and development of three additional lesions. Figure E reveals bilateral periventricular plaques and a small right peripheral lesion. Figure F demonstrates a significant enlargement of plaques at the same level and three to four new lesions.

He presented to our emergency department three weeks after initial diagnosis with complaints of significant weakness, multiple falls, shortness of breath, dysarthria, and worsening paresthesia. A CT scan of the head at this time showed a 1.8 cm lesion within the posterior limb of the right internal capsule, consistent with white matter disease. The patient was admitted to the neurology service and started on 125 mg/2L Solumedrol. Lumbar puncture revealed elevated protein, elevated IgG and albumin with an increased ratio, and 10 CSF oligoclonal bands. PCR testing was negative for HHV-6 DNA at this time. MRI of the brain showed extensive new white matter plaques throughout (Figure 1). MRI spine showed new and worsening demyelinating lesions in the cervical and thoracic spine. After finishing his course of Solumedrol, the patient was discharged with a close follow-up appointment in the neurology clinic. During this visit, he endorsed an improvement in overall strength but continued numbness in his hands and feet bilaterally. He endorsed pain radiating to the spine and arms with head movement. Physical exam showed intact cranial nerves but brisk reflexes on the right compared to the left. His sensation was intact and his gait was steady with the use of a walker. At the clinic visit, he was started on 100 mg gabapentin three times daily and 600 mg IV ocrelizumab infusions every 5-6 months. The patient will continue to follow our neurology team and receive treatment for MS.

## Discussion

Herpesviruses possess the ability to invade the central nervous system (CNS) through several processes. A frequently proposed mechanism involves retrograde migration from peripheral neurons into the CNS. HHV-6 is primarily transmitted via respiratory secretions, and this virus has been found in olfactory nervous tissue [4]. It is hypothesized that the connection between the olfactory nerve and the nasal passage via septal olfactory nerves provides a direct route for infection [4]. Another theorized mechanism is hematogenous spread by overcoming the blood-brain barrier via latent infection of white blood cells. The known affinity of HHV-6 for CD4 T-cells and the herpesviruses defining ability for dormant survival and reactivation provides a reasonable hypothesis for this route of CNS infection [4].

MS has been historically described as an autoimmune disease attributable to cross-reactive CD4 T-cells causing demyelination of CNS neurons. One study supports this theory, showing that these T-cells have specific reactivity to MBP in MS patients compared to controls [5]. A differentiated subset of naïve CD4 T-cells called Th17 cells has also been implicated in this process [5]. A proinflammatory cytokine, IL-17, produced by these cells has been the focus of research investigating the demyelination process. When secreted, IL-17 causes an influx of white blood cells and other synergistic inflammatory mediators [5]. Studies show that Th17 cells readily cross the blood-brain barrier, that IL-17 mRNA levels are high and correlate to active disease in MS patients, and that Th17 cells are more numerous during a relapse compared to controls [5]. Finally, IL-17 is shown to cause direct death of oligodendrocytes, the glial cell lineage that is responsible for myelinating CNS neurons [6].

Many studies have examined the relationship between HHV-6 activity and the progression of established MS. Early literature provided evidence that the worsening of MS disease burden was correlated with HHV-6 antibodies or DNA in serum samples [7,8]. However, a recent prospective cohort study did not find significant data to indicate HHV-6 immunoglobulin activity has an association with MS exacerbation [9]. A 2017 meta-analysis included studies that collected blood, serum, CSF, peripheral blood mononuclear cells, tissue, and saliva specimens to detect infection. Only nine of these 42 studies collected CSF samples and it was not specified if the data was collected in the acute or relapse phase of the disease [10]. The FilmArray® meningitis encephalitis CSF panel (BioFire Diagnostics, Salt Lake City, UT, USA), used at both hospitals in our case, provides a 90% sensitivity and 97% specificity for infection [11].

Our observation is that inconsistent work-up of MS-like disease processes has contributed to the absence of a clear definition of the role of HHV-6 in acute MS presentation. To best determine if HHV-6 is correlated with the onset of MS, a lab test with the narrowest window of detection should be used. Meningitis encephalitis panel provides real-time detection of infection through amplification of viral DNA. This type of study is most accurate when samples are taken between three days and two weeks of symptom onset [12]. IgM levels in serum are detectable as early as seven days and as late as three months. This creates a challenge in accurately determining the acute period of infection [13]. As exemplified in this case, the timeline between diagnosis and relapse was only three weeks. Thus, it is possible for our patient to be seropositive for HHV-6 IgM in both the acute and relapse period, making it difficult to determine the virus’ role in MS disease. In this case, quantification of viral DNA in CSF allowed us to determine that HHV-6 was present at diagnosis but not during relapse.

Most studies rely on immunoglobulin levels to determine the presence of infection. Thus, it is important to determine if this is an effective method of analysis. Because HHV-6 is commonly contracted by children at a young age, many adults have generated immunoglobulins to this virus [13]. This obscures the interpretation of positivity for anti-HHV6 IgG present in blood samples. Due to a lengthy period between infection and IgG positivity, this method of analysis is not appropriate for determining acute infection of HHV-6 [13].

This case highlights the need for more rapid identification of HHV-6 infection to determine the virus’ role in MS disease. We emphasize that using serum IgM and IgG titers to illustrate the presence of infection has many limitations. CSF analysis by lumbar puncture using PCR quantification of viral DNA allows for the most time-sensitive detection. Although there is conflicting literature concerning HHV-6 causing relapse, its role in the etiology of MS has yet to be investigated.

## Conclusions

This case is significant for the novel identification of HHV-6 viral DNA in CSF fluid upon initial presentation of MS. Current literature focuses on the virus’ association with MS relapse, however, there is a lack of data concerning the acute presentation period. Thus, a prospective cohort study that analyzes CSF via PCR at the time of diagnosis of MS could further elucidate the role that HHV-6 infection plays in the development of MS. 
